# A cross-sectional analysis of persistent low back pain, using correlations between lumbar stiffness, pressure pain threshold, and heat pain threshold

**DOI:** 10.1186/s12998-021-00391-4

**Published:** 2021-09-03

**Authors:** Casper Glissmann Nim, Søren O’Neill, Anne Gellert Geltoft, Line Korsholm Jensen, Berit Schiøttz-Christensen, Gregory Neil Kawchuk

**Affiliations:** 1 Spine Center of Southern Denmark, University Hospital of Southern Denmark, Oestrehougvej 55, 5500 Middelfart, Denmark; 2 Department of Regional Health Research, University of Southern Denmark, Campusvej 55, 5230 Odense M, Denmark; 3 Department of Physical Therapy, University of Alberta, 8205 114St, 2-50 Corbett Hall, Edmonton, AB T6G 2G4 Canada

**Keywords:** Low back pain, Mechanics, Hyperalgesia, Central sensitization

## Abstract

**Introduction:**

Little is known about the underlying biomechanical cause of low back pain (LBP). Recently, technological advances have made it possible to quantify biomechanical and neurophysiological measurements, potentially relevant factors in understanding LBP etiology. However, few studies have explored the relation between these factors. This study aims to quantify the correlation between biomechanical and neurophysiological outcomes in non-specific LBP and examine whether these correlations differ when considered regionally vs. segmentally.

**Methods:**

This is a secondary cross-sectional analysis of 132 participants with persistent non-specific LBP. Biomechanical data included spinal stiffness (global stiffness) measured by a rolling indenter. Neurophysiological data included pain sensitivity (pressure pain threshold and heat pain threshold) measured by a pressure algometer and a thermode. Correlations were tested using Pearson’s product-moment correlation or Spearman’s rank correlation as appropriate. The association between these outcomes and the segmental level was tested using ANOVA with post-hoc Tukey corrected comparisons.

**Results:**

A moderate positive correlation was found between spinal stiffness and pressure pain threshold, i.e., high degrees of stiffness were associated with high pressure pain thresholds. The correlation between spinal stiffness and heat pain threshold was poor and not statistically significant. Aside from a statistically significant minor association between the lower and the upper lumbar segments and stiffness, no other segmental relation was shown.

**Conclusions:**

The moderate correlation between spinal stiffness and mechanical pain sensitivity was the opposite of expected, meaning higher degrees of stiffness was associated with higher pressure pain thresholds. No clinically relevant segmental association existed.

## Introduction

The lumbar spine is a complex anatomical structure, the chief function of which is biomechanical—to bear loads through various static and dynamic functions and provide protection for soft neural tissue [1]. However, it is not apparent from patient history, clinical examination, or diagnostic imaging when perturbations in biomechanical function are causal factors for developing low back pain (LBP), when they result from LBP, and when they are simply irrelevant normal variants [2]. Hence, LBP is often considered non-specific. In fact, only around 5–10% of LBP can be attributed to an explicit patho-anatomical issue. In the remaining 90–95%, there is no apparent structural issue [3]. In a clinical setting among manual therapy providers, it is common to attribute such non-specific LBP to permutations in biomechanical function, i.e., as a causal factor, although the evidence is lacking [2, 4, 5].

Much research has been conducted to understand LBP’s etiology better, but progress has been limited [6–8]. Arguably, this is the result of limitations in measurement, methodology, and population sampling. In measurement, recent technological advances have made it possible to collect data in new areas, which could shed light on LBP’s underlying causes. In particular, the development of new technologies to quantify spinal stiffness non-invasively [9], thus better quantifying what is thought to be an influential clinical factor in LBP [10]. Spinal stiffness has shown promise in that it may be associated with treatment-induced disability improvements. E.g., in patients with LBP, those who have immediate reductions in spinal stiffness after spinal manipulation have improvements in disability (≥ 30% reduction in The Oswestry Disability Index), and this change in stiffness does not occur for those who do not have improvements in disability [11, 12].

In parallel to exploring the mechanical aspect of LBP, researchers are also exploring the underlying mechanisms of the pain experience itself. This includes quantitative sensory testing (QST), which quantifies individual pain perception in response to controlled noxious stimuli [13]. Such experimental tests can differentiate LBP patients from healthy controls, and perturbations in pain modulation (sensitization of the somatosensory system e.g., leading to decreases of pain thresholds) appear to manifest in the sub-acute stage as pain turns persistent [14]. It is of interest that these perturbations, in a commonly noted mechanical syndrome such as LBP, extend beyond deep muscle mechanical pain sensitivity, i.e., pressure pain, to superficial skin measures, e.g., heat pain. Nevertheless, apparent differences have previously been reported for both these measurements between LBP patients and healthy controls [15, 16].

Furthermore, each new technology allows researchers to explore these properties at the segmental level [9, 15], an important consideration considering that back pain is often thought to be localized to specific anatomic areas pertaining to a given segmental level. While biomechanical testing has been used to evaluate the spine’s primary biomechanical function, and experimental QST has done the same for neurophysiological function, it seems unlikely that the two are not interconnected. We put forward that another factor that may hinder our understanding of LBP etiology is artificial segregation of biomechanics and neurophysiology. These two aspects of LBP are often discussed, studied, and treated in research and clinical settings as if they are distinct phenomena, when in reality, they may well be interconnected [17]. Studying biomechanical and neurophysiological systems together may provide important information to understand the etiology of LBP better. This is particularly important in the field of manual therapy, where the treatment site is often determined using a mixture of stiffness and pain locations [17]. However, we know little about the segmental interplay between stiffness and pain sensitivity.

Hence, this study will use an experimental test setup that mimics clinical practice to investigate the relationship between lumbar stiffness and mechanical and non-mechanical QSTs, two measurements that appear to correlate with each other [18]. Yet, whether a correlation also exists with mechanical spinal stiffness is unknown. Furthermore, all three experimental tests appear to be affected following manual therapy [12, 19, 20].

Therefore, the specific aims of this investigation are to (1) quantify the correlation between biomechanical and neurophysiological measurements (global stiffness, pressure pain threshold, and heat pain threshold) in LBP patients, and (2) examine if these correlations differ when considered regionally (the lumbar back) or segmentally (e.g., L4).

From a clinical standpoint, we hypothesize that stiffness and pain sensitivity are negatively associated, i.e., high degrees of stiffness and low pain threshold are correlated, and that this association may be greater for (1) a mechanical stimuli as opposed to a thermal stimuli and (2) segmentally versus regionally.

## Methods

### Design

Our study design was a secondary observational cross-sectional analysis of baseline data from a randomized trial of participants with persistent non-specific LBP [21]. The study was approved by the Regional Committee on Health Research Ethics for Southern Denmark (S-20160201). The manuscript was prepared in reference to the STROBE format.

### Setting

A population sample of patients seen at *the Spine Center of Southern Denmark*, a public, regional hospital department specializing in spinal pain, was recruited consecutively between November 2017 and February 2019.

### Participants

Participants were included based on a diagnosis of non-specific LBP from a clinician at the Spine Center. A total of 132 participants were included using the following criteria [21]:

- Persistent non-specific LBP.
- No surgical indication or previous spinal surgery.
- Daily oral opioid intake was limited to 40 mg of morphine at the time of inclusion.
- Body mass index under 35 kg/m^2^.
- Age between 18 and 60 years old.

### Procedure

All testing was done at the Spine Center by one rater who gained experience with the test procedures through practical laboratory training, including pilot testing on 20 participants with persistent LBP not included in the present study.

The baseline test session was initiated by identifying each lumbar segment. Each spine process from S1 to T12 was marked superficially with a marker with the participant in the prone position. The segment identification was confirmed using ultrasonography (Sonosite Titan Linear, L38 probe) [22].

Afterward, we completed the protocol in the following standardized order for all participants, deep mechanical pain sensitivity, superficial thermal pain sensitivity, and spinal stiffness with sufficient rest time of approximately two minutes between each procedure, limiting potential interactions. Each procedure is described below in greater detail.

#### Spinal stiffness

Spinal stiffness was tested using the VerteTrack (VT). The device consists of two weighted probe wheels (3 cm apart) that slowly rolls along the lumbar paravertebrales guided by the surface markings of spinous process locations with the subject in a prone position. The landing site is centered around the S1 spine process and the lifting site at the T12 spine process. The resulting posterior-to-anterior displacement during rolling is measured by a string potentiometer, which can then be quantified as stiffness (applied mass/displacement or N/mm) with a sampling rate of 30 Hz. This process is then repeated with increasing loads of 10 N up to a maximum of 60 N. Before testing, each participant was instructed to exhale and hold their breath at around the residual air volume while completely relaxing their muscle until the trial was complete. If pain or discomfort were elicited, the procedure would be repeated one more time, and if the trial continued to produce discomfort, the procedure was discontinued. Only trials with no discomfort were used for the analysis as trials with discomfort could lead to muscle guarding and erroneous stiffness measures. The VT is a novel experimental device reported as comfortable and safe [23], has good reliability in asymptomatic subjects [9], and demonstrates high accuracy under bench-top conditions [24]—suggesting that the VT can be used to assess spinal stiffness in-vivo.

#### Deep mechanical pain sensitivity

Deep mechanical pain sensitivity was determined using a pressure algometer (Model 2, Somedic, Sweden) with a custom-made double-headed probe (2 × 1 cm^2^, 3 cm apart), which allowed for bilateral pressure at either side of the mid-line for each lumbar segment. The pressure was increased gradually with an approximate rate of 50 kPa/s until the participant reported the pressure as painful by pressing an indicator button. If no pain had been elicited by 1000 kPa, the test was discontinued, and 1000 kPa was recorded as the pressure pain threshold (PPT). The pressure algometer has excellent intra-rater reliability for patients with LBP [25].

#### Superficial thermal pain sensitivity

Superficial thermal pain sensitivity was assessed using a handheld thermode (Medoc TSA-II, Israel) with a single 30 × 30 mm probe placed in the midline for each lumbar segment centered at the spinous process ensuring complete contact between skin surface and probe. The baseline temperature was pre-set to 32 degrees Celsius (C). During testing, the temperature increased at a rate of 1 C/s until the participant reported the temperature as painful by pressing an indicator button. Maximum temperature was pre-set at 50 C, and if no pain had been elicited by then, this was recorded as the heat pain threshold (HPT). Using the thermode to indicate the HPT has good-to-excellent intra-rater reliability when tested at the spine of asymptomatic volunteers [26].

All QST tests (PPT and HPT) were performed, with the patient in the prone position, at each spinal segment three times. The segments were tested in a pre-determined, computer-generated, random order with 10-s rest intervals between each test. If no pain had been elicited by 1000 kPa after the first two trials, a third trial was not performed for PPT. Before data collection, one or two test trials of each pain threshold assessment were performed at the lower extremity and one test at the T12 segment to familiarize participants with the procedure.

### Variables of interest

**Fig. 1:**
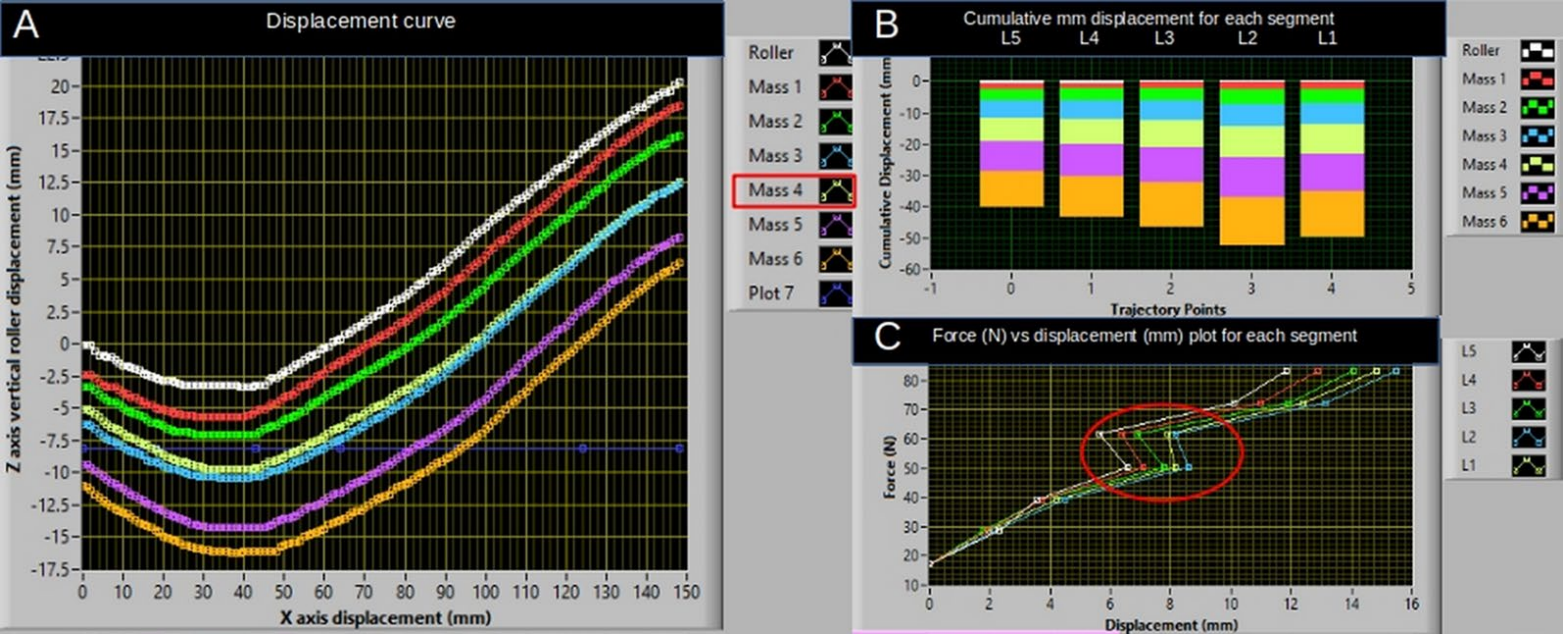
An example of the LabView output. **A** The displacement curve, the y-axis represent vertical displacement for each trajectory point along the lumbar lordosis (x-axis). Each load trajectory is presented as a unique color (each trial with a different load). **B** The cumulative displacement (mm) for each segment across loads. **C** The force (N) versus displacement curve (mm) for each segment. In this example, we would omit the light-yellow 40 N line (mass 4) as the displacement recorded was less than that recorded with 30 N (mass 3), suggesting muscle guarding. This is also illustrated by the skewness of the plot in (**C**) (red circle). After data cleaning, the stiffest segment was L5 with a global stiffness score of 5.5, and the least stiff segment was L2 with a global stiffness score of 4.2 (**B**)

Lumbar stiffness: The VT data were smoothed (SVD algorithm, polynomial order of 2, least-squares method, and tolerance of 0.0001) and visualized (Labview 15.0f3 for Windows 10, National Instruments, Texas, USA) before being exported to a spreadsheet (LibreOffice, vers. 7, for Ubuntu 18.04) for further analysis. Global lumbar stiffness (GS) indicated stiffness throughout the available load and was calculated as the slope of force–displacement (N/mm) from the second load to the second highest load. Hence, the terminal loads were removed (e.g., 0 N and 60 N). As part of the data analysis, a subjective inspection of the smoothed data was completed before extracting the data. Some loads within the participant trial were affected by factors such as breathing, muscle guarding, or technical errors and were omitted. This process was guided by visual inspection of the displacement curve corresponding to each load. See Fig. 1 for a LabView output and an example of a removed trial. Global stiffness is a continuous parameter ranging from 0 to $\infty$ and was recorded for each segment [L1–L5].

Deep muscle pain sensitivity: Pressure pain threshold is a continuous parameter ranging from 0 to 1000 kPa and was calculated for each segment [L1–L5] as the average of three trials.

Superficial skin pain sensitivity: Heat pain threshold is a continuous parameter ranging from 32 to 50 C and was calculated for each segment [L1–L5] as the average of three trials.

### Statistical analysis

#### Descriptive statistics

Descriptive data, including the demographics and all outcomes, are presented as means, medians, standard deviations, and interquartile ranges. Normal distribution for each outcome was visualized using density and QQ-plots. Visual inspection for skewness and data shape was further conducted [27].

#### Correlation

The correlation between the three outcomes is presented visually as Loess slopes [28] plotted for each segment. Pearson’s product-moment correlation (ρ) was used for parametric data, and Spearman’s rank correlation (R_s_) was used for non-parametric data. We omitted individual participants if they did not have data for both the parameters in question. The strength of the correlations was evaluated as *poor* (< 0.30), *moderate* (0.30 ≤ 0.50), *good* (0.51 ≤ 0.70) and *strong* (> 0.70) [29]. All correlations were examined as a single summarized value for all segments for each participant and individually for each segment. A *p* value < 0.05 was considered to be significant.

#### Segmental statistics

Segmental data are depicted as means and 95% confidence intervals. The association between outcomes was tested using a one-way analysis with the outcomes as the dependent variable and segment as the independent variable. The assumptions for the ANOVA were tested for (1) normality by plotting the residuals against predicted values and (2) homogeneity of variances using Levene’s test. A *p* value of less than 0.1 would indicate further post-hoc testing using Tukey multiple pairwise comparisons to investigate between-segment differences. The results for each outcome are presented as F-statistics, between-segment difference and adjusted *p* values.

Data analyses were completed using R [30], for Linux, v. 4.0 with R-studio v. 1.4. Data cleaning were performed using the Tidyverse [31].

## Results

### Participants characteristics

Of the 132 participants included, complete experimental baseline data were available for 128 (3 participants had missing HPT data, 1 participant had incomplete GS data). The sample consisted of 72 (55%) males. Complete demographic data are presented in Table 1.

**Table 1:** A demographic profile of the 132 participants included in the analysis

Parameter	Mean	SD	Median	IQR
Age	45.1	9.7	46.0	14.2
Body Mass Index	26.2	3.9	25.6	4.9
Low back pain intensity	5.6	1.8	5.5	2.7
Oswestry disability index	27.8	11.6	27.3	18.5
Pain duration (months)	48.2	78.1	14.8	53.9

All data were normally distributed except for PPT. None of the variables were visually skewed and overall had consistent data shape across outcomes. All experimental outcomes are presented in Table 2 as a single average score for all segments and each segment. None of the participants reached the maximum of 1000 kPa or 50 °C.

**Table 2:** The regional experimental outcome measurements of the 132 participants included in the analysis

Outcome	Segment	Mean	SD	Median	IQR
Global stiffness	All	4.14	0.88	4.05	1.13
	L1	4.01	0.88	3.89	0.94
	L2	3.97	0.86	3.90	0.97
	L3	4.03	0.84	3.93	0.92
	L4	4.20	0.85	4.11	1.06
	L5	4.48	0.90	4.40	1.07
Pressure pain threshold	All	475	231	446	363
	L1	522	244	523	369
	L2	482	228	465	357
	L3	472	225	452	376
	L4	455	225	437	346
	L5	445	229	401	338
Heat pain threshold	All	42.2	3.7	42.0	5.6
	L1	42.5	3.6	42.4	5.3
	L2	42.3	3.8	42.4	6.3
	L3	42.0	3.6	41.3	5.3
	L4	42.1	3.8	41.6	5.6
	L5	42.0	3.6	41.8	5.7

### Correlation data

**Fig. 2:**
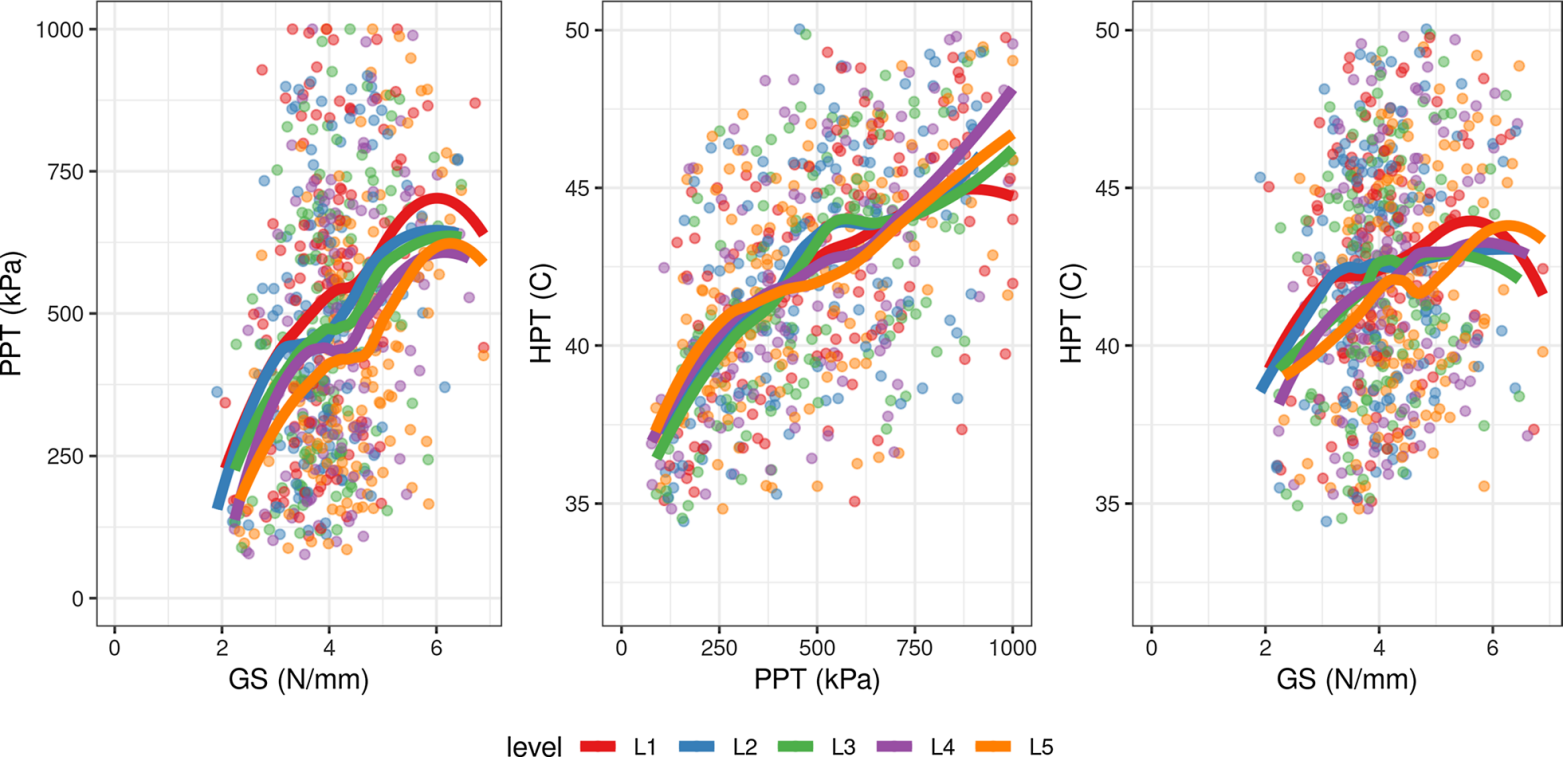
Correlation between lumbar stiffness, mechanical pain sensitivity, and superficial pain sensitivity, presented as Loess slopes for each segment. *GS* global stiffness, *PPT* pressure pain threshold, *HPT* heat pain threshold

The relationship between segments and each outcome is depicted in Fig. 2. Visually the correlation does not differentiate between segments.

Table 3 lists the correlations and *p* values for all outcomes. All correlations were statistically significant, except for the correlation between GS and HPT at the L2 segment (*p* value = 0.12). All correlations had a positive direction. The correlation between GS and PPT for all segments was moderate (ρ = 0.38). Conversely, the correlation between GS and HPT was poor (R_s_ = 0.23) and not significant. The correlations between PPT and HPT were good for all segments (ρ = 0.53).

**Table 3:** Correlation for all and every segment between lumbar stiffness, pressure pain, and superficial pain sensitivity

	Global stiffness versus Pressure pain threshold		Global stiffness versus Heat pain threshold		Pressure pain threshold versus Heat pain threshold	
Segment	R_s_	*p* value	ρ	*p* value	R_s_	*p* value
All	0.38	< 0.01	0.23	< 0.05	0.58	< 0.01
L1	0.36	< 0.01	0.19	0.03	0.53	< 0.01
L2	0.33	< 0.01	0.14	0.12	0.55	< 0.01
L3	0.34	< 0.01	0.21	< 0.05	0.57	< 0.01
L4	0.33	< 0.01	0.22	< 0.05	0.56	< 0.01
L5	0.38	< 0.01	0.28	< 0.01	0.50	< 0.01

Presented as chi^2^ scores and *p* values

R_s_ = Spearman’s rank correlation, ρ = Pearson’s product-moment correlation

### Segmental data

**Fig. 3:**
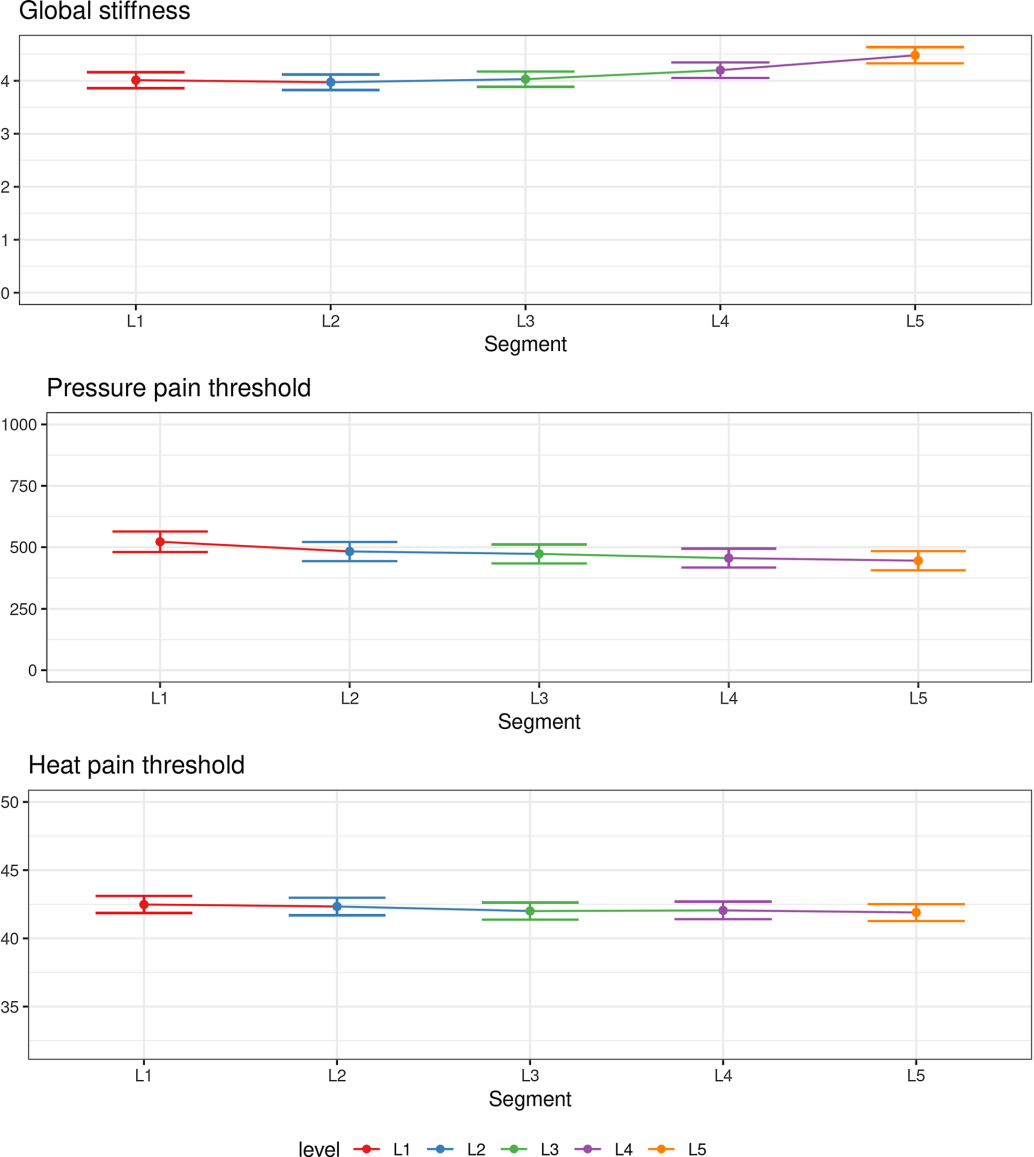
The segmental value for lumbar stiffness (global stiffness—N/mm), deep pressure pain (pressure pain threshold—kPa), and superficial pain (heat pain threshold—°C). Connected means with error bars indicating the 95% confidence interval

All outcomes are presented visually in Fig. 3. A segmental pattern can be observed from L1 to L5. Global stiffness increased while PPT and HPT decreased caudally. This indicates that L5 is the stiffest segment and the most sensitive segment for both pressure and heat pain thresholds. The between-segment difference in GS and HPT for the upper segments is almost negligible. All model assumptions were upheld for the ANOVA. The ANOVA revealed the following: There was a statistically significant effect of spinal segmental level on GS (F4,650 = 7.7, p < 0.01). Whereas, we found no significant effect on PPT (F4,655 = 2.2, p = 0.07) or HPT (F4,640 = 7.7, p = 0.68). This indicates that a statistically significant association was apparent for GS, while PPT had a *p* value of just above 0.05. Heat pain threshold was clearly not significant and exceeded the cut point (*p* value = 0.1). Post-hoc testing of GS and PPT were, consequently, indicated.

For GS, a significant adjusted difference was observed in the Tukey multiple comparisons for L5 against L1, L2, and L3. No segments differed significantly for the PPT scores when considering the adjusted scores. However, as Fig. 3 demonstrates, L5 and L4 have the lowest PPT scores compared to L1. All values are available in Table 4.

**Table 4:** Post hoc Tukey comparison after ANOVA testing of lumbar stiffness and pressure pain threshold between each segment

Segment	Global stiffness		Pressure pain threshold	
	Difference (95% CI)	Adj. *p* value	Difference (95% CI)	Adj. *p* value
L2–L1	− 0.04 (− 0.33 to 0.25)	~ 1	− 39.5 (− 117.2 to 38.3)	0.63
L3–L1	0.02 (− 0.27 to 0.31)	~ 1	− 49.6 (− 127.3 to 28.2)	0.41
L4–L1	0.19 (− 0.10 to 0.48)	0.40	− 66.7 (− 144.5 to 11.0)	0.13
L5–L1	**0.47 (0.18 to 0.76)**	**< 0.01**	− 77.0 (− 154.8 to 0.7)	0.05
L3–L2	0.06 (− 0.23 to 0.35)	0.98	− 10.10 (− 87.9 to 67.7)	~ 1
L4–L2	0.23 (− 0.06 to 0.52)	0.21	− 27.24 (− 105.0 to 50.5)	0.87
L5–L2	**0.51 (0.22 to 0.80)**	**< 0.01**	− 37.6 (− 115.3 to 40.2)	0.68
L4–L3	0.17 (− 0.12 to 0.46)	0.50	− 17.14 (− 94.9 to 60.6)	0.97
L5–L3	**0.45 (0.16 to 0.74)**	**< 0.01**	− 27.46 (− 105.2 to 50.3)	0.87
L5–L4	0.28 (− 0.01 to 0.57)	0.07	− 10.32 (− 88.1 to 67.4)	~ 1

Bold indicates a significant adjusted statistical difference between segments

## Discussion

### Summary of the results

Our results demonstrated a *moderate* positive correlation between GS and PPT; higher stiffness scores were associated with higher deep mechanical pain thresholds. In contrast, the correlation between GS and HPT was poor, while, as expected, a good correlation was observed between deep and superficial pain thresholds. When examining stiffness and pain sensitivity across segments, we only observed a statistically significant difference in stiffness between higher and lower lumbar segments.

### Correlation findings

Surprisingly, the correlation observed between spinal stiffness and pressure pain threshold was opposite than expected: Participants with higher degrees of spinal stiffness also had higher pressure pain thresholds (i.e., lower pain sensitivity). Three different postulations could view this relation: (1) The increased lumbar stiffness might be explained as part of an adaptive mechanical protection system [32] that decreases nociceptive activity in the lumbar region and therefore increases PPT. This is consistent with similar research. When inducing pain at the low back in two asymptomatic populations, higher degrees of stiffness were observed [33], whereas the PPT score did not change [34]. Possibly, and opposed to pain sensitivity, stiffness could be viewed as a continuum where high degrees of stiffness can be advantageous for the locomotor system. (2) This could be reversed, so higher pain thresholds increase stiffness again as a protective adaption. (3) Possibly, as a perceptual influence—a stiffer spine may be perceived as more resilient to applied forces. Given that pain is considered a protective response, a stiffer spine might require less protection, resulting in an increased ability to tolerate force (i.e., higher PPTs). In contrast, in a less stiff spine, the spine may be perceived as less resilient to applied forces, with low PPT scores through psychological mediation (increased protection needed). However, the authors are not aware of any research that has investigated this previously for LBP. Nevertheless, there is evidence of this connection between mind and body in prior work from other fields [35].

A *good* correlation observed between PPT and HPT is congruent with previous findings [18]. This correlation may simply reflect shared modulation of both central as well as peripheral pain mechanisms. This is an interesting finding as these measurements differ within the aspect of pain processing. A mechanical pressure involves activating deep tissue afferent fibers and thermal stimuli involving peripheral skin activation [36].

### Segmental findings

Figure 3 revealed an apparent minor association between all the outcomes, which reached statistical significance for lumbar stiffness. When comparing outcomes between segments, a difference in mean stiffness was observed between the higher and the lower lumbar segments. However, the largest mean difference in stiffness observed was between L5 and L2 and corresponded to 11%, which is only marginally higher than the mean manual detectable threshold of change in stiffness at 8% [37]. The small difference between the largest values suggests it is nearly impossible to palpate differences in stiffness between closer or adjacent segments. Furthermore, this difference is possibly even smaller as the current analysis did not consider the standard error of measurement for the VT [38].

For the QST, no differences were found between segments. This is consistent with a prior study conducted at our laboratory [15]. Arguably, this is due to the sample’s chronicity, indicating that the original nociceptive input has developed into a generalized peripheral sensitization [39]. Furthermore, changes at the supra-spinal level could also lead to the generalized effect observed for both thresholds. Current research indicates that persistent LBP patients often have perturbations in neurological mapping of the somatosensory system and cortical homunculus or “*cortical smudging*” [40]. Potentially, such cortical smudging could lead to difficulty identifying the different stimuli at the nearby segments. This argument is supported by previous findings of distorted body images [41] and difficulty identifying the midline of the trunk under painful sensorimotor manipulation in persistent LBP patients [42]. Also, another study investigated the neural activity of the hemisphere, and when testing PPT at L1 and L5 segments in healthy subjects, an activity overlap of 76% was observed at the right hemisphere and 59% at the left hemisphere [43]. Theoretically, this area of activity is likely to increase with pain chronicity, as persistent LBP patients also have difficulty extending beyond 2-point-discrimination to decreased graphesthesia at the lower back compared to healthy controls [44]. It is also possible that the spatial resolution of painful sensory input is too poor in the lumbar region to differentiate one segment from another.

The previously described cortical smudging could also affect movement behaviors such as postural control [45], lumbopelvic motor control [46], and thoracolumbar dissociation [40]. This indicates that patients with persistent LBP are probably less able to perceive lumbar stiffness reliably. This is highlighted by the findings of Stanton et al., who reported that patients with persistent LBP felt significantly stiffer compared to healthy controls. However, when measuring lumbar stiffness using mechanical indentation, no between-group difference was observed [47]. Additionally, in a similar cohort of LBP patients, self-reported stiffness was not associated with two different stiffness measures obtained from the VT [48]. Suggesting that perceived stiffness may genuinely be a perceptual influence. Whether this finding is present in acute LBP is unknown.

### Methodological considerations

A strength of the study was the large sample size compared to our previous topography study [15], albeit the measures were limited to the midline. All tests were conducted by the same rater limiting the intra-rater variability. While the VT is computer-controlled, further minimizing the risk of rater-based errors, this was not the case for the QST. We endeavored to reduce the risk of bias through multiple training sessions, allowing the participants up to three test stimuli to familiarize themselves with the procedures. Furthermore, both QST measurements are commonly used and, thus, vigorously described and tested in the literature [13, 49, 50].

However, the stiffness measurement was a single plane indentation and possibly not a clinically relevant measure of stiffness. It is not yet known if mechanical measurements of stiffness are clinically relevant beyond a few studies [11, 12, 21]. Manual palpation has some benefits over the experimental testing used in this study. It is possible to direct the pressure for stiffness and pain in multiple planes, examine trophic changes of the skin and muscles, perform joint-play, locate non-verbal reflectory muscle guarding and tender points using verbal feedback. The evidence for these factors is arguably sparse, and manual palpation carries with a considerable risk of bias [51]. Other biomechanical factors may better reflect spinal biomechanics such as local muscle activity [52], multifidus thickness, or disc diffusion [12]. Finally, the VT has only been deemed reliable in an asymptomatic population, and we currently do not have evidence that demonstrates the same measurement properties apply to LBP patients. However, the reliability score is consistent with the single indentation device, a similar technique, examined on LBP patients [38].

Another limitation is the lack of data on the most symptomatic clinical segment. While not apparent in this analysis, this localized point could potentially provide a more meaningful correlation, which we currently miss in the averaged data. Finally, this was a cross-sectional study that did not compare to other LBP groups or healthy controls. Thus the results are, therefore, only applicable for secondary care persistent non-specific LBP patients.

## Conclusion

The a-priori hypothesis could not be confirmed. We found moderate correlations between spinal stiffness and mechanical pain sensitivity to be the opposite of what we expected; higher degrees of stiffness were associated with higher pressure pain threshold. As suspected, pressure and heat pain thresholds had a good correlation, while stiffness and heat sensitivity were poorly correlated. We observed no clinically relevant lumbar between-segment association for any of the outcomes in this population sample of persistent non-specific low back pain patients.

## Data Availability

Data is available upon reasonable request. Please contact casper.nim@rsyd.dk.

## Abbreviations

LBP: Low back pain
QST: Quantitative sensory test
VT: VerteTrack
PPT: Pressure pain threshold
C: Celsius
HPT: Heat pain threshold
GS: Global lumbar stiffness
ρ: Pearson’s product-moment correlation
R_s_: Spearman’s rank correlation
